# Survey of treatment and care practices in small-cell lung cancer among German radiation oncologists

**DOI:** 10.1007/s00066-022-02019-9

**Published:** 2022-11-23

**Authors:** J. Gnüchtel, D. Vordermark, D. Medenwald

**Affiliations:** Universitätsklinik und Poliklinik für Strahlentherapie, Martin-Luther-Universität Halle-Wittenberg, Halle, Germany

**Keywords:** Radiotherapy, Chemotherapy, Immunotherapy, Prophylactic cranial irradiation, Atezolizumab

## Abstract

**Background:**

The management of small-cell lung cancer shows differences, particularly with regard to the use of radio- (RT), chemo-, and immunotherapy. We performed a survey among German radiation oncologists to assess the management of small-cell lung cancer (SCLC).

**Methods:**

A 34-question online survey was created and sent out by e‑mail to radiation oncologists throughout Germany. The survey period extended from August 2020 to January 2021. The questions addressed indications for RT, planning techniques, dosing/fractionation, target volume definition for consolidative thoracic irradiation, and the use of prophylactic cranial irradiation (PCI). At the same time, we surveyed the use of atezolizumab. The survey addressed the treatment practice for limited-stage SCLC (LS-SCLC) and extensive-stage SCLC (ES-SCLC).

**Results:**

We received 74 responses. In LS-SCLC, treatment is planned predominantly based on diagnostic information from computed tomography (CT) of the thorax/abdomen/pelvis (88%), PET-CT (86%), and pulmonary function testing (88%). In LS-SCLC, 99% of respondents perform radiation concurrently with chemotherapy, preferably starting with cycle one or two (71%) of chemotherapy. The most common dose and fractionation schedule was 60–66 Gy in 30–33 fractions (once daily: 62% of all respondents). In ES-SCLC, 30 Gy in 10 fractions (once daily: 33% of all respondents) was the most commonly used regimen in consolidative thoracic irradiation. Only 25% use chemosensitization with RT. The inclusion criteria for PCI were similar for limited and extensive disease, with Karnofsky index (78% and 75%) being the most important decision factor. Respondents use a schedule of 30 Gy in 15 fractions most frequently in both stages (68% limited stage [LS], 60% extensive stage [ES]). Immunotherapy was used regularly or occasionally in LS-SCLC by 45% of respondents, with reduced lung function (37%), cardiac comorbidities (30%), and hepatic insufficiency (30%) being the most commonly mentioned exclusion criteria for this form of therapy. In ES-SCLC, atezolizumab use was reported in 78% of all questionnaires. Half of the respondents (49%) administer it simultaneously with cranial irradiation.

**Conclusion:**

Our survey showed variability in the management of SCLC. Results from future studies might help to clarify open questions regarding the optimal treatment paradigms. In addition, new treatment modalities, such as immunotherapy, might change practices in the near future.

## Introduction

Lung cancer is one of the most common malignant cancers in Germany, with small-cell carcinoma accounting for approximately 16% of all cases [1]. It is characterized by aggressive growth and early development of metastases.

Although standard therapy consisting of chemotherapy and radiation has been shown to be effective, there continues to be wide variation in treatment and care practices.

The importance of chemotherapy is reflected by both limited and extensive disease stages. Several studies demonstrated the survival benefit of using chemotherapy compared to surgery alone [2].

In addition, there remains a conflict regarding the use of prophylactic cranial irradiation (PCI). For example, what is the benefit of PCI for patients who have been radiologically proven to be free of brain metastases after chemoradiotherapy (CRT) [3]? In addition, which dosage and fractionation is most effective? Furthermore, there are discrepancies regarding the inclusion criteria and which patient population benefits most from this form of therapy [4]. Previous studies also investigated to which extent and at what intervals control examinations should be performed as follow-up, especially in patients in whom no prophylactic cranial irradiation was performed [3].

New treatment methods, such as antibody therapy with the programmed cell death ligand 1 (PD-L1) atezolizumab, are coming more into focus and have already been able to establish themselves in practice to some extent [5].

Based on the CASPIAN trial [6], another PDL1-ligand, durvalumab, was approved for treatment of SCLC in 2021 [7], but this was only after our survey was performed. Therefore, with regard to checkpoint inhibitors, our survey included only atezolizumab, which was already approved in 2019 [8].

## Methods

We compiled a survey of 34 items, which we sent out by e‑mail to about 1300 radiation oncologists throughout Germany (Table 3). The survey period extended from August 2020 to January 2021 and we received a total of 74 responses.

The questions were initially related to demographic data. To identify the location of the participants’ practices, we divided the states into four groups: region north: Bremen, Hamburg, Mecklenburg-Western Pomerania, Lower Saxony, and Schleswig-Holstein; region west: Hesse, North Rhine-Westphalia, Rhineland-Palatinate, and Saarland; region east: Berlin, Brandenburg, Saxony, Saxony-Anhalt, and Thuringia; region south: Baden-Württemberg and Bavaria.

In addition, we asked for the physician’s approximate age, years of professional experience, and the number of patients with SCLC cared for annually. The survey was conducted anonymously, and we were only able to obtain a more detailed analysis based on these demographic data. Our project was evaluated for ethical clearance and received approval from the ethics committee of the Medical Faculty, Martin Luther University Halle-Wittenberg (reference number 2020-139).

The main part of the survey was related to management of LS-SCLC and ES-SCLC. The radiation oncologists were asked to select the answers that most closely matched their standard of care in each case.

The primary focus of the questions was use of consolidative thoracic irradiation and prophylactic cranial irradiation. We evaluated fractionation and dosing, planning techniques, definition of the target volume, and patient-specific inclusion criteria, among other factors.

We also considered the use of antibody therapy with atezolizumab.

## Results

### Demographics

**Fig. 1:**
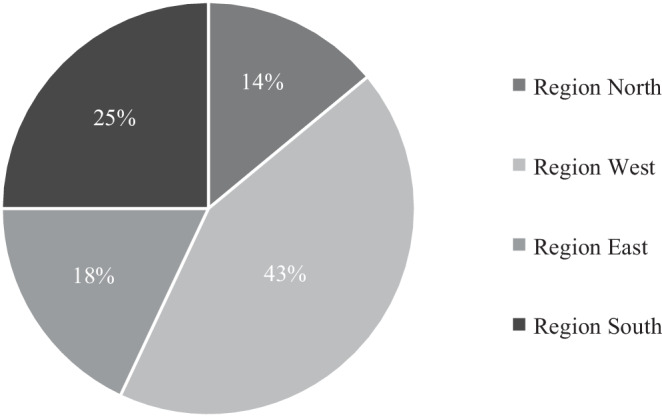
Regional distribution of respondents

We received responses from all four of the regional subgroups, with region west being the most represented at 43%, followed by region south at 25%, and region east and region north at 18 and 14%, respectively (Fig. 1).

Participant age varied widely, from 20–29 years to > 60 years, although every age group was represented. In addition, differences in professional experience were evident. The number of lung cancer patients treated by a radiation oncologist per year ranged from 5 to 300, 42 physicians (58%) reported that their proportion of SCLC patients was 11 to 20%.

### LS-SCLC

We asked radiation oncologists which diagnostic methods they generally use for treatment planning. With more than 85% each, CT of the thorax/abdomen/pelvis (88%), PET-CT (86%), a planning CT with dosimetric limits (85%), and pulmonary function testing (88%) were reported most frequently. Seventy-five percent also mentioned using EBUS or mediastinoscopy as a diagnostic tool. Cranial magnetic resonance imaging was reported as another important method.

#### Management of radiotherapy in LS-SCLC

**Fig. 2:**
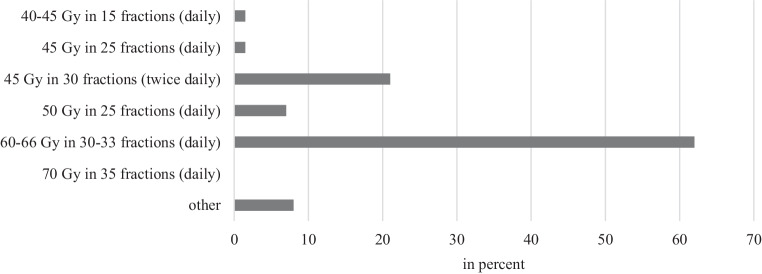
Dosage and fractionation in simultaneous radiochemotherapy for limited-stage small-cell lung cancer

Reportedly, the most commonly used schedule for thoracic irradiation was 60 to 66 Gy in 30 to 33 fractions once a day (62%). In 22%, 45 Gy in 30 fractions BID (twice a day) was used (Fig. 2).

Ninety-nine percent of the radiation oncologists reported to apply radiation simultaneously with chemotherapy; 1% sequentially due to poor tolerability. The initiation of RT was most frequently indicated in the first or second cycle of chemotherapy (71%), much less frequently in the third or fourth cycle (27%).

Concerning the clinical target volume at the beginning of the second cycle in patients with T2N2M0 LS-SCLC, 33% defined it as macroscopic tumor volume including contiguous lymph node stations and an additional margin. Tumor volume with additional margins to consider microscopic involvement (30%) was mentioned to a similar extent.

If tumor volume decreases after the first chemotherapy cycle, 41% of the respondents would leave the target volume unchanged according to the planning CT and 21% would expand it to the pretherapeutic volume. A compromise between both would be chosen by 38%.

For positional control in radiotherapy, 65% would use kilovoltage cone-beam CT, 33% would use megavoltage cone-beam CT. Most participants indicated daily use of image guidance (51%). Twelve percent reported daily application for the first few days and once a week during further treatment (Table 1).

**Table 1:** Positional control and frequency of image guidance in radiotherapy of limited-stage small-cell lung cancer

**Positional control**	**Responses, *n*** **(%)**
Kilovoltage orthogonal	17 (23.6)
Megavoltage orthogonal	11 (15.3)
Kilovoltage cone-beam CT	47 (65.3)
Megavoltage CT/cone-beam CT	24 (33.3)
Other	4 (5.6)
**Interval of image guidance**	
Daily	37 (50.7)
Weekly	20 (27.4)
Other	16 (21.9)

#### PCI in LS-SCLC

**Fig. 3:**
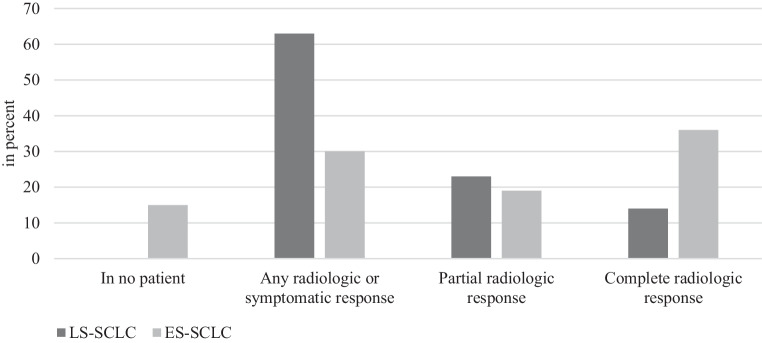
Use of prophylactic cranial irradiation in relation to radiologic or symptomatic response after radiochemotherapy in limited-stage small-cell lung cancer (*LS-SCLC*) and extensive-stage small-cell lung cancer (*ES-SCLC*)

In the majority of cases (63%), PCI would be used in patients with any radiologic or symptomatic response to chemoradiotherapy; in 14% it would only be used in patients with complete radiologic response (Fig. 3).

Among these factors, the Karnofsky index (78%) was the most important in deciding whether to offer cranial irradiation. In addition, cognitive ability (44%) was also an important consideration. Patient age over 70 years would influence the decision in only 4% (Table 2).

**Table 2:** Overview of responses regarding factors affecting the decision to use PCI in limited and extensive stages

Variable	Responses, *n* (%)	
	LS-SCLC	ES-SCLC
Clinical and radiological response	7 (9.6)	5 (6.9)
Extent of primary tumor	9 (12.3)	11 (15.3)
Karnofsky index or performance status	57 (78.0)	54 (75.0)
Significant weight loss (> 10–15%)	7 (9.6)	8 (11.1)
Toxicity of radiochemotherapy	26 (35.6)	21 (29.2)
Use of extrathoracic consolidative irradiation	–	6 (8.3)
Use of consolidative irradiation of intrathoracic manifestation	–	18 (25.0)
Basic cognitive ability	32 (43.8)	34 (47.2)
No metastases in repeated cranial imaging	26 (35.6)	26 (36.1)
Age	3 (4.2)	1 (1.4)
Compliance	1 (1.4)	–
Comorbidities	1 (1.4)	1 (1.4)

*n* number, *LS-SCLC* limited stage small-cell lung cancer, *ES-SCLC* extensive stage small-cell lung cancer

**Fig. 4:**
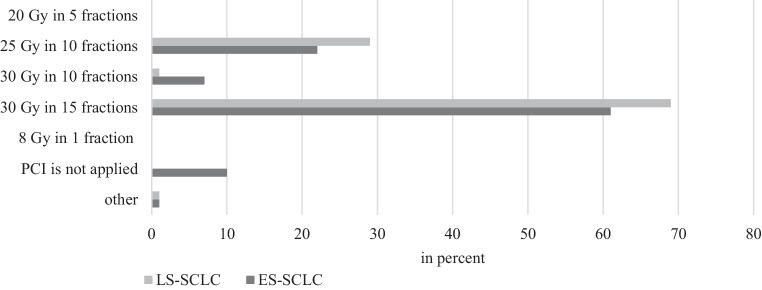
Dosage and fractionation in prophylactic cranial irradiation for limited-stage small-cell lung cancer (*LS-SCLC*) and extensive-stage small-cell lung cancer (*ES-SCLC*)

A dose of 30 Gy in 15 fractions (68%) was the most common schedule for PCI, followed by 25 Gy in 10 fractions (29%; Fig. 4).

#### Antibody therapy with atezolizumab in LS-SCLC

**Fig. 5:**
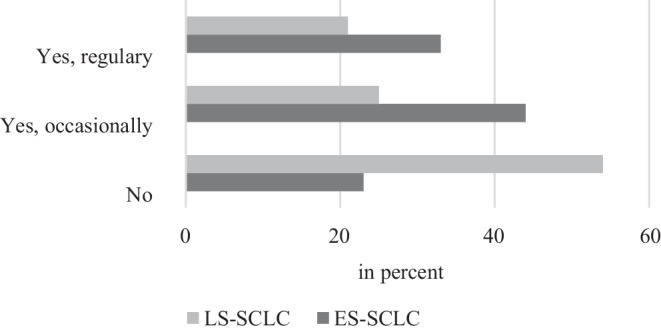
Frequency of use of antibody therapy with atezolizumab in limited-stage small-cell lung cancer (*LS-SCLC*) and extensive-stage small-cell lung cancer (*ES-SCLC*)

Less than half (45%) of the respondents would use antibody therapy with atezolizumab in the limited disease stage (Fig. 5).

Exclusion criteria for this therapy were mainly reduced lung function (37%), cardiac comorbidities (30%), or liver insufficiency (30%). A cut-off age was not specified.

#### Special situations in LS-SCLC

The radiation oncologists were asked whether a patient with contralateral supraclavicular lymph node involvement would be irradiated. Nearly 1/3 (30%) indicated that this patient had extensive disease by definition, and that irradiation would not be used in this case. Fifteen percent would use irradiation routinely, more than half (55%) only when dosimetrically safe to do so.

For a patient in a clinical T1/2a N0 LS-SCLC stage, the majority (51%) indicated radiotherapy to be a primary intervention simultaneous with chemotherapy. Otherwise, it would be used as adjuvant treatment postoperatively and with a pN2 situation (25%) or regardless of pathologic status (14%).

### ES-SCLC

#### Management of radiotherapy in ES-SCLC

**Fig. 6:**
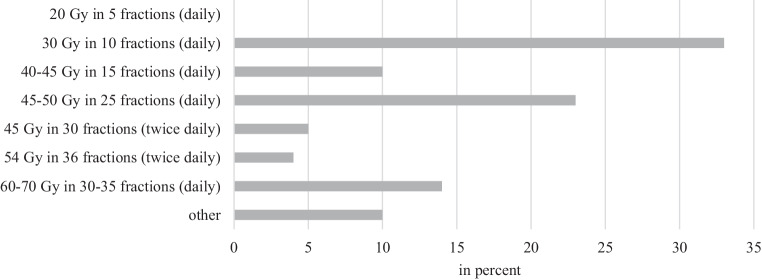
Dosage and fractionation in simultaneous radiochemotherapy for extensive-stage small-cell lung cancer

All participants reported offering radiation to all symptomatic patients with ES-SCLC. When asked whether thoracic radiotherapy was relevant after chemotherapy, the majority (56%) reported that it would be used for any response, and insofar as the thoracic tumor burden was the greatest. Thirty percent would use it when there was a complete radiologic response to chemotherapy outside the thorax and a complete or partial response within the thorax. The most commonly used schedules were 30 Gy in 10 fractions once a day (33%) and 45–50 Gy in 25 fractions once a day (23%; Fig. 6). Furthermore, 25% indicated a simultaneous chemosensitization with consolidative radiation.

Tumor remnants plus prechemotherapy-involved parenchymal foci and lymph node stations were most commonly used to define the clinical target volume in ES-SCLC (42%). Slightly less frequently (33%), only the tumor remnants would represent the target volume.

Consolidative radiotherapy outside the thorax would be given by 65% of the participants, with the majority (61%) assuming a limited tumor before and after chemotherapy. Oligometastasis defines tumor limitation in this context.

#### PCI in ES-SCLC

Most of the respondents answered that they would perform PCI if there was a complete (35%) or partial (19%) radiologic response to chemotherapy. Fifteen percent of radiation oncologists reported that cranial irradiation would not be used at all in patients with ES-SCLC (Fig. 3).

Similar to the decision factors in LS-SCLC regarding whether to use PCI, the clinical performance according to ECOG or Karnofsky index (75%) and cognitive ability (47%) were the most frequently mentioned parameters in extensive disease. Confirmation of the absence of brain metastases on cranial control imaging (36%) was indicated as another important factor (Table 2).

A schedule with 30 Gy in 15 fractions (60%) was most common (Fig. 4).

#### Antibody therapy with atezolizumab in ES-SCLC

Thirty-three percent of respondents reported regular use of atezolizumab in patients with ES-SCLC, while 45% would use it occasionally. Nearly one fifth (22%) would not use this therapeutic modality (Fig. 5). About half of the respondents (49%) mentioned that simultaneous use with PCI would not be an issue. Otherwise, minimum intervals of 1 day (14%) or 1 week (29%) were indicated.

## Discussion

The survey showed that differences in SCLC care practices are relevant in German radiotherapy centers. This refers to application regimens in radiation, indications for radiation, or even the use of antibody therapy.

### LS-LSCS

A large consensus existed in terms of overall treatment planning, with wide application of PET-CT for target volume definition alongside conventional CT. For staging and disease recurrence, FDG-PET-CT plays a major role due to its high sensitivity and specificity. Detection of distant metastases and delineation of the primary tumor and lymph nodes allow optimal planning of radiation. For NSCLC, an international randomized multicenter trial [9] has already shown that the use of FDG-PET in treatment planning reduced CTVs. In multimodality protocols, metabolic FDG-PET parameters (i.e., maximum standardized uptake value [SUV_max_], metabolic tumor volume [MTV]) [9] were found to significantly correlate with overall and progression-free survival, and/or local tumor control [9]. PET tracers other than FDG have been investigated in lung carcinoma. Hypoxia markers in non-small-cell lung cancer (NSCLC) predicted poor outcome and thus could be helpful in planning treatment escalation [9].

Furthermore, in simultaneous chemoradiotherapy, treatment regimens vary, with 66 Gy in 30 to 33 fractions (once daily) and 45 Gy in 30 fractions (twice daily) mentioned most frequently in the survey (Fig. 2).

In the randomized CONVERT trial by Faivre-Finn et al. [10], these two regimens were compared in terms of overall survival (OS). Median OS was 30 months (95% confidence interval [CI] 24–34) in the twice-daily group versus 25 months (21–31) in the once-daily group [10]. Similarly, there was a non-significant benefit in 2‑year OS (56% vs. 51%) for the twice-daily regimen. Adverse events related to grade 3 to 4 esophagitis occurred with similar frequency (19% vs. 19%); neutropenia occurred more frequently in the 66 Gy in 30 fractions group (74% vs. 65%). The study found that there were only minor differences between the two regimens in terms of OS [10]. Hyperfractionation was found to be a reasonable approach to treat SCLC in terms of duration and compliance, but is not the preferred schedule in clinical routine.

The Norwegian THORA trial [11] compared high-dose thoracic irradiation of 60 Gy in 40 fractions with a standard dose of 45 Gy in 30 fractions, with both groups of patients receiving irradiation twice per day. The 2‑year OS showed a significant advantage on the side of high-dose irradiation (74% vs. 48%). Increased toxicity was not observed in the 60 Gy group. Thus, according to the authors, this treatment regimen should be considered as an alternative [11].

According to our survey, radiotherapy was most often started in cycle one or two of chemotherapy. Murray et al. [12] compared early radiation parallel to the first cycle with late radiation parallel to the last cycle of chemotherapy. An advantage of the early group in comparison to the late group was found in terms of progression-free survival (PFS; 15.4 vs. 11.8 months) and OS (21.2 vs. 16 months) [12].

De Ruysscher et al. [13] supported this approach in their meta-analysis. They compared OS in early (according to the study inclusion criterion, before cycle three of chemotherapy) and late radiotherapy onset, with early onset showing an absolute survival gain of 7.7% at 5 years compared with a later start. It should be mentioned that an early onset led more often to development of acute severe esophagitis (odds ratio [OR] 1.93 [1.45–2.56]) [13].

Hu et al. [14] compared hyperfractionated RT (1.5 Gy twice a day in 30 fractions) and hypofractionated RT (2.5 Gy once a day in 22 fractions) to evaluate the influence of the timing of RT in addition to efficacy. Good median OS (28.3 vs. 22.0 months) and locoregional control (LRC; 1‑year LRC 82.1% vs. 60.7%, 2‑year LRC 84.9% vs. 68.8%) were achieved in both groups. Using a Cox regression analysis, it was found that time from chemotherapy initiation to RT less than 43 days was associated with better LRC. Similarly, a period of less than 63 days between the start of chemotherapy and the end of RT was associated with improved OS. It was concluded that timing is more important than dose intensification [14].

#### PCI LS-SCLC

A meta-analysis of Yin et al. [15] published in 2019 included seven randomized controlled trials (RCT) from 1987 to 2017 to compare overall survival and the incidence of brain metastases in patients with PCI vs. without PCI. It showed that the use of PCI resulted in a significantly reduced incidence of brain metastases and slightly prolonged OS. It should be noted that patients who received cranial imaging following CRT and were free of brain metastases had no OS benefit from PCI (hazard ratio [HR] = 0.94; 95% CI 0.74–1.18). In contrast, patients without imaging showed a significant benefit (HR = 0.70; 95% CI 0.57–0.85) [15].

To further investigate the development of brain metastases in LS-SCLC, Levy et al. [16] used the data from the CONVERT trial [10]. The authors examined whether there was an association between the incidence of brain metastases and the application schedule of thoracic irradiation. In this trial, 82% of all patients received subsequent PCI after CRT, with 8% of previously once-daily and 9% of previously twice-daily thoracic irradiated patients developing brain metastases. Thus, the type of thoracic irradiation was found to have no effect on brain metastasis [16].

Zheng et al. [17] identified risk factors for the development of brain metastases in patients with LS-SCLC without PCI. Retrospectively, they found that high T stage, high neutrophil-to-lymphocyte ratio, early thoracic radiotherapy, and fewer chemotherapy cycles increased the risk of developing brain metastases [17].

#### Atezolizumab LS-SCLC

One third of our respondents reported regular use of atezolizumab in patients with LS-SCLC, 44% occasionally. While regular use was reported in 33% of respondents from region west, it was significantly less in the east (15%) and south (11%) regions. In region north, no regular use was described.

A phase II/III randomized trial by Higgins et al. [18] initiated in May 2019 is evaluating the combination of chemotherapy and atezolizumab in patients with LS-SCLC. Primary endpoints are PFS and OS. Results are expected in 2024. Atezolizumab is expected to yield a hazard ratio of 0.62 for PFS and 0.71 for OS at a one-sided significance level of 0.025 [18].

In the following we have compared our survey results and the abovementioned studies with the current national guidelines [25] for the treatment of SCLC. The guideline recommends use of combined chemotherapy with cisplatin and etoposide, which should be used over four to six cycles. Ninety-nine percent of our respondents use cisplatin/etoposide. According to the guideline [25], radiotherapy should be used in all patients with tumor extension that can be irradiated. This should be simultaneous to chemotherapy and be started early. According to our survey, 71% of radiation oncologists start radiotherapy in cycle one or two, 27% in cycle three or four of chemotherapy. In the CONVERT study [10], no significant difference between the use of a conventional treatment regimen with 60–66 Gy in 30 fractions (once daily) and a hyperfractionated regimen with 45 Gy in 30 fractions (twice daily) could be demonstrated. Based on this, the German guideline [25] recommends both regimens. This was also evident in our survey. Sixty-two percent of the respondents treat with 60–66 Gy in 30 fractions (once daily) and 21% with 45 Gy in 30 fractions (twice daily).

The guideline recommends PCI in all patients in remission [25]. According to our survey, 63% use PCI for any response to chemotherapy, 23% for partial radiologic response, and 14% for complete radiologic response. A regimen of 25 or 30 Gy in 2.0 or 2.5 single doses, respectively, is recommended; this is confirmed by 99% of our respondents.

Regarding antibody therapy, there are no recommendations in the current guideline. This was reflected in our survey. The frequency of use of atezolizumab therapy varied widely. As mentioned above, studies [18] are currently being conducted on this topic. More precise recommendations must be based on these results.

### ES-SCLC

#### PCI ES-SCLC

Slotman et al. [19] published an RCT in 2007 in which they evaluated the efficacy of PCI in patients with ES-SCLC and any response to chemotherapy. The risk of brain metastasis was 14.6% in the PCI group and 40.4% in the control group. The 1‑year survival rate was 27.1% with PCI and 13.3% without intervention [19]. Because this trial failed to include imaging in the form of MRI before study entry and the brain metastasis status was therefore unknown, the validity of this study has been questioned.

In this regard, a Japanese RCT by Takahashi et al. [3] investigated the efficacy of PCI in patients with extensive-stage SCLC in whom brain metastases could be excluded by MRI prior to study entry and compared OS between the PCI group and the observation group. A control MRI was performed at 3‑monthly intervals for 12 months and at 18 and 24 months thereafter. The study found that the risk of brain metastasis at 12 months was 32.9% in the PCI group and 59% in the observation group. The OS, however, was 11.6 months in the PCI group and 13.7 months in the observation group. The authors concluded that PCI is not necessary in patients who are free of brain metastases provided there is follow-up with regular controls every 3 months for 1 year and at 18 and 24 months thereafter. Asymptomatic metastases then should be treated with radiotherapy and chemotherapy [3].

A 2018 meta-analysis [20] also examined the role of PCI in ES-SCLC, showing an advantage of the PCI group over the non-PCI group in terms of 1‑year survival (37.1% vs. 27.1%), PFS (HR = 0.83; 95% CI 0.70–0.98), and risk of brain metastasis (HR = 0.34; 95% CI 0.23–0.50). OS specifically improved in patients younger than 65 years. However, no significant OS benefit was observed overall [20].

Chen et al. [21] studied the effect of early vs. late PCI. Early PCI was defined as an interval between the initiation of chemotherapy and the initiation of radiation of less than 6 months and late PCI as an interval of more than 6 months. Primary endpoint was the incidence of brain metastases, which was significantly lower in the early PCI group than in the late PCI group (HR, 0.45; 95% CI 0.23–0.89; *p* = 0.024) [21].

#### Atezolizumab ES-SCLC

According to our survey, immunotherapy with atezolizumab was used regularly in 33% and occasionally in 44%, while it did not significantly feature in the clinical routine of 23% of the respondents.

In the west of Germany, 37% of radiation oncologists reported regular use of atezolizumab, slightly less in the southern (33%) and eastern (30%) regions. As in LS-SCLC, this form of therapy was least common in the northern region (20%).

The IMPower133 RCT [5] evaluated the combination of atezolizumab and carboplatin plus etoposide in patients with ES-SCLC. Endpoints represented overall survival and progression-free survival. Compared with the placebo group, the atezolizumab group had significantly increased OS (12.3 vs. 10.3 months; HR 0.70; 95% CI 0.54 to 0.91; *p* = 0.007) and prolonged PFS (5.2 vs. 4.3 months). Relative to individual baseline characteristics, the combination with immunotherapy also showed benefits. Thus, patients younger than 65 years (OS 12.1 months atezolizumab vs. 11.5 months placebo) and older than 65 years (12.5 vs. 9.6 months) benefited. This relation was similar for patients with an ECOG score of zero (16.6 vs. 12.4 months) and one (11.4 vs. 9.3 months). Patients with brain metastases showed an advantage in the placebo group (8.5 vs. 9.7 months). The study included patients with brain metastases, while no differences in OS or PFS were observed. Nevertheless, due to the small population, further studies are necessary to establish standards in the treatment of patients with brain metastases and immunotherapy. With regard to side effects, mainly in the form of neutropenia, anemia, thrombocytopenia, alopecia, and nausea, the combination of etoposide and atezolizumab showed no difference compared to chemotherapy alone (grade 1 or 2 36.9 vs. 34.7%, grade 3 or 4 56.6 vs. 56.1%, grade 5 both 1.5%) [5].

Mansfield et al. [22] also examined atezolizumab therapy in terms of a risk–benefit profile. For this purpose, adverse events from the IMpower133 trial [5] and subjective patient assessments were evaluated. Health-related quality of life improved in both groups, but the improvement was significantly more pronounced and long-lasting in the atezolizumab group [22].

Atezolizumab is approved for patients with ES-SCLC but is also used in limited disease. The extent to which there is a benefit from immunotherapy at this stage is unclear, due to the paucity of data at this point. As noted above, Higgins et al. [18] are evaluating the survival benefit and progression-free survival with atezolizumab in patients with LS-SCLC, and results are expected in 2024 [18].

There is also an issue with its use in patients with brain metastases. These were included in the IMpower trial [5], but in a small population, so results from subgroups will need to follow in the future. To evaluate different immunotherapies, Zhou et al. [23] published a meta-analysis in 2019 that compared chemotherapy alone and chemotherapy in combination with a PD-L1 antibody, CTLA‑4 antibody (cytotoxic T‑lymphocyte-associated protein 4), or VEGF antibody (vascular endothelial growth factor). Here, the combination with atezolizumab showed the greatest benefit for OS (12.3 vs. 10.3 months) and no increased toxic effects compared to etoposide–platinum therapy alone (58.1% vs. 57.7%). Bevacizumab, a monoclonal antibody against VEGF, showed the best PFS but did not confer an OS advantage (8.9 vs. 9.8 months). At the same time, it was associated with the highest rate of treatment-related adverse events (TRAE) (62.1% vs. 54.7%). Similarly, the combination of etoposide–platinum plus ipilimumab showed no significant difference for OS (11 vs. 10.9 months) [23].

The CASPIAN trial [6], conducted between 2017 and 2018, evaluated the efficacy of the PDL1 ligand durvalumab in combination with platin–etoposide compared to platin–etoposide alone. It showed a significant improvement in median OS for durvalumab (13.0 vs. 10.3 months) [6]. Also examined was the combination of durvalumab plus the CTLA-4-antibody tremelimumab and platin–etoposide vs. platin–etoposide alone. There was no benefit in terms of OS (10.4 vs. 10.5 months) and thus no evidence for synergistic effects of these two immunotherapies [24].

Regarding the dosage and fractionation of radiotherapy in ES-SCLC, no recommendations are found in the current national guideline [25]. The answers in our survey were also very varied in this respect: 33% apply 30 Gy in 10 fractions, 23% 45–50 Gy in 25 fractions, and 14% 60–70 Gy in 30–35 fractions. Accordingly, a recommendation would result in more homogeneous application.

The guideline states that patients with a response to first-line chemotherapy should be offered PCI or have regular MRI checks every 3 months in the first year. A regimen of 25 or 30 Gy in 2.0 or 2.5 Gy single doses, respectively, is recommended [25]; 83% of our respondents applied it this way.

The national guideline specifically recommends combining chemotherapy with immunotherapy. In addition to the abovementioned studies, meta-analyses clearly showed that patients benefited from PD-L1 checkpoint inhibitor therapy regardless of age or performance status [25]. However, in our survey, antibody therapy was used regularly in only 33% and occasionally in 44%. Although there is a clear recommendation for combined chemotherapy and immunotherapy, its use is not yet established in many centers according to our survey. More precise recommendations are needed because of the variability in the use of radiotherapy.

A limitation of the survey is the low number of participants. With 74 questionnaires answered, we had a response rate of less than 1%. However, it should be mentioned here that we sent the survey to individual radiation oncologists. We assume that in many cases the questionnaires were answered on behalf of a clinic or medical practice. However, it is also possible that several radiotherapists from the same institution answered the questionnaire. Because our survey was anonymous, we do not have data on the type of facility from which the responses originated. Therefore, we cannot infer whether there are differences in terms of care between university hospitals, non-university hospitals, and the ambulatory setting.

Of course, a survey as a method of data collection also brings disadvantages. Due to the multiple-choice system, survey participants are bound to answers, even though we allowed an open response for several questions.

## Conclusion

Our survey showed that German radiation oncologists have the same standards in many approaches, but that there are differences in certain treatment methods. Dosing, fractionation, and inclusion criteria for RT and PCI continue to have no clear consensus, although survival benefits for certain regimens have been noted in some published trials. The survey also showed variable frequency of use of immunotherapy with atezolizumab. More results are needed to further establish this form of therapy in both stages. Thus, we hope that in the future, through additional trials, treatment and care practices can be better standardized and new therapeutic methods established.

## Appendix

**Table 3:** Survey

1. Do you treat lung cancer with radiation therapy? □ Yes □ No
*Demographics*
2. Please tell us your approximate age. □ 20–29 □ 30–39 □ 40–49 □ 50–59 □ > 60
3. In which region do you currently practice? □ Region North (Bremen, Hamburg, Mecklenburg-Western Pomerania, Lower Saxony, Schleswig-Holstein) □ Region West (Hesse, North Rhine-Westphalia, Rhineland-Palatinate, Saarland) □ Region East (Berlin, Brandenburg, Saxony, Saxony-Anhalt, Thuringia) □ Region South (Baden-Württemberg, Bavaria)
4. For approximately how many years have you been treating lung cancer? □ < 5 years □ 5–10 years □ 10–15 years □ 15–20 years □ > 20 years
5. How many newly diagnosed lung cancer patients do you treat per year? _______
6. How many of these patients reported in question 5 have a diagnosis of SCLC? □ < 10% □ 11–20% □ 21–30% □ > 30%
*Limited disease of SCLC*
7. What diagnostic methods do you use to plan treatment for an LS-SCLC? Please select all that apply. □ CT chest, abdomen, pelvic □ PET-CT □ EBUS/mediastinoscopy □ Pleura cytology □ X-Ray □ CT simulation in dosimetric constraints □ Pulmonary function testing (FEV1, DLCO) □ Other (please specify): ______
8. Would you irradiate a patient with contralateral supraclavicular lymph node involvement? Please select the answer that is closest to your standard of care. □ No, because by definition this patient is classified as having extensive disease according to prospective randomized trials. □ Yes, but only if it is dosimetrically safe to perform □ Yes, routinely
9. How would you describe your initial radiotherapy regimen for a patient in a clinical T1/2a N0 SCLC stage? Please select the answer that is closest to your standard of care. □ As a primary treatment simultaneous to chemotherapy (without surgery) □ As adjuvant treatment postoperatively, regardless of pathological status □ As adjuvant treatment postoperatively, if the lymph node status is pN2
10. In most cases of LS-SCLC, simultaneous radiochemotherapy is the current standard of care. What is your current dose and fractionation preference for radiotherapy? Please select the answer that is closest to your standard of care. □ 40–45 Gy in 15 fractions (once daily) □ 45 Gy in 25 fractions (once daily) □ 45 Gy in 30 fractions (twice daily) □ 50 Gy in 25 fractions (once daily) □ 60–66 Gy in 30–33 fractions (once daily) □ 70 Gy in 35 fractions (once daily) □ Other (please specify): ______
11. Do your patients with LS-SCLC most often receive combined chemotherapy of a platinum derivative (cisplatin or carboplatin) and etoposide (46 cycles) simultaneously with radiotherapy? If “No,” please indicate the regimen which is used. □ Yes □ If “No,” please specify your regimen: ______
12. During which cycle of chemotherapy do you prefer to start concurrent radiochemotherapy (CRT) in patients with LS-SCLC? Please select the answer that is closest to your standard of care. □ Cycle 1 or 2 □ Cycle 3 or 4 □ Cycle 5 or 6 □ Radiation is given after chemotherapy (not simultaneously, but sequentially) □ The choice of cycle generally does not matter. □ The choice of cycle does not matter as long as the total treatment time does not exceed 30 days AND platinum-based chemotherapy is given
13. When planning radiochemotherapy for a patient with T2N2M0 LS-SCLC at the start of the second cycle of chemotherapy, how is the clinical target volume (CTV) typically defined for you? Please select the answer that is closest to your standard of care. □ The macroscopic tumor volume (GTV) alone as presented in the current planning CT (no additional margin in the sense of a CTV) □ Macroscopic tumor volume with additional margins to account for microscopic involvement □ Macroscopic tumor volume including adjacent lymph node stations and an additional margin □ Macroscopic tumor volume plus the ipsilateral mediastinum, the ipsilateral hilus, and an additional margin □ Macroscopic tumor volume plus the entire mediastinum, the ipsilateral hilum, and an additional margin
14. If the tumor volume at the time of planning CT decreases after a first cycle of chemotherapy, would you expand the clinical target volume to the tumor volume that existed before chemotherapy? □ Yes, I would include the entire pretherapeutic volume. □ No, my clinical target volume would not change, regardless. □ I would choose something in between
15. What image-based methods do you use for positional control in radiotherapy of a LS-SCLC? □ Kilovoltage orthogonal □ Megavoltage orthogonal □ Kilovoltage cone beam CT □ Megavoltage CT/Cone-Beam CT □ Others (please specify): ______
16. At what intervals do you use an image guidance? □ Only at the beginning of treatment □ Weekly □ Daily □ Other (please specify): ______
17. In which patient with LS-SCLC are you most likely to use prophylactic cranial irradiation (PCI) after radiochemotherapy? □ In no patients □ Patients with any response to radiochemotherapy (radiologic or symptomatic) □ Patients with a partial radiologic response to radiochemotherapy □ Patients with a complete radiologic response
18. Are there other factors that would affect your decision to offer PCI at LS-SCLC? Please select all that apply. □ Not applicable, as I do not use PCI at LS-SCLC □ No, clinical and radiological response are the most important factors □ Extent of primary tumor (bulky disease) □ Karnofsky index or performance status □ Significant weight loss (> 10–15%) □ Toxicity of radiochemotherapy □ Basic cognitive ability □ Repeated cranial imaging (CT, MRI) shows no metastases □ Other (please specify): ______
19. What dose and fractionation do you use for PCI in LS-SCLC? □ 20 Gy in 5 fractions □ 25 Gy in 10 fractions □ 30 Gy in 10 fractions □ 30 Gy in 15 fractions □ 8 Gy in 1 fraction □ Not applicable, as I do not use PCI with LS-SCLC □ Other (please specify): ______
20. How do you plan to perform PCI? Please select the answer that is closest to your standard of care. □ Clinical only setting with or without thermoplastic mask □ Virtual simulation with or without mask □ Complete CT simulation with mask □ CT simulation with mask and hippocampal avoidance □ Not applicable, as I do not use PCI for LS-SCLC □ Other (please specify): ______
21. Do your patients use atezolizumab in combination with platinum-based chemotherapy as part of limited disease radiochemotherapy? □ Yes, regularly □ Yes, occasionally □ No, not in the stage of limited disease
22. Do you have any criteria that would lead you to abstain from atezolizumab therapy? Please select all possible answers (if used in the stage of limited disease in the context of chemotherapy outside the actual radiation phase). □ Impaired lung function □ Cardiac comorbidity (NYHA) □ Kidney dysfunction (eGFR) □ Liver insufficiency (Child score) □ Current smoker □ Regardless of smoking status □ Brain metastases □ No brain metastases □ Independent of brain metastases □ Cut-off age (if so, please enter the age): ______
*Extensive disease of SCLC*
23. Do you offer radiation therapy to relieve symptoms in patients with symptomatic ES-SCLC? □ Yes □ No
24. Do you consider consolidative thoracic radiation relevant after palliative chemotherapy? Please select the answer that is closest to your standard of care. □ No □ I use consolidative thoracic radiotherapy only in the context of clinical trials. □ Yes, only if there is a complete radiologic response to chemotherapy outside the thorax and a complete or partial response inside the thorax. □ Yes, for any response to chemotherapy when thoracic manifestation is the major tumor burden □ Yes, for any response to chemotherapy, regardless of tumor burden □ Other (please specify): ______
25. What dose and fractionation do you use for consolidative thoracic irradiation? □ 20 Gy in 5 fractions (once daily) □ 30 Gy in 10 fractions (once daily) □ 40–45 Gy in 15 fractions (once daily) □ 45–50 Gy in 25 fractions (once daily) □ 45 Gy in 30 fractions (twice daily) □ 54 Gy in 36 fractions (twice daily) □ 60–70 Gy in 30–35 fractions (once daily) □ Not applicable, as I do not use consolidative irradiation for ES-SCLC. □ Other (please specify): ______
26. Would you use simultaneous chemosensitization with consolidative radiation? □ Yes □ No □ Not applicable, as I do not use consolidating irradiation
27. What would be your clinical target volume for consolidative irradiation of an ES-SCLC? Please select the answer that is closest to your standard of care. □ Only tumor residues as visualized on planning CT □ Tumor residues and the parenchymal foci and lymph node stations involved prior to chemotherapy □ Tumor residues and the entire mediastinum □ Not applicable, as I do not use consolidative radiotherapy. □ Other (please specify): ______
28. Do you use consolidative radiation outside the thorax in patients with ES-SCLC? Please select the answer that is closest to your standard of care. □ No □ Yes, only in the context of a clinical trial □ Yes, only if tumor was limited before and after chemotherapy (oligometastatic) □ Yes, only if the tumor was limited after chemotherapy (oligometastatic), regardless of the situation before chemotherapy
29. In which patient with ES-SCLC are you most likely to use prophylactic cranial irradiation after palliative chemotherapy? Please select the answer that is closest to your standard of care. □ In no patient □ Patients with any response (radiologic or symptomatic) to chemotherapy □ Patients with a partial radiologic response to chemotherapy □ Patients with a complete radiologic response to chemotherapy
30. Are there other factors that would affect your decision to use PCI at ES-SCLC? Please select all that apply. □ Not applicable, as I do not use PCI at ES-SCLC. □ No, clinical and radiological response are the most important factors □ Karnofsky index or performance status □ Size of primary tumor in relation to distant metastases □ Use of consolidative irradiation of intrathoracic manifestation □ Use of extrathoracic consolidative irradiation □ Significant weight loss (> 10–15%) □ Toxicity of chemotherapy □ Cognitive function at the beginning □ Repeat cranial imaging (CT, MRI) shows no metastases □ Other (please specify): ______
31. What dose and fractionation do you use for PCI in ES-SCLC? □ 20 Gy in 5 fractions □ 25 Gy in 10 fractions □ 30 Gy in 10 fractions □ 30 Gy in 15 fractions □ 8 Gy in 1 fraction □ Not applicable, as I do not use PCI with ES-SCLC □ Other (please specify): ______
32. How do you plan to perform PCI? Please select the answer that is closest to your standard of care. □ Clinical only setting with or without thermoplastic mask □ Virtual simulation with or without mask □ Complete CT simulation with mask □ Complete CT simulation with mask and hippocampal avoidance □ Not applicable, as I do not use PCI for LS-SCLC □ Other (please specify): ______
33. Do you use atezolizumab in combination with platinum-based chemotherapy as part of extensive disease radiochemotherapy in your patients? □ Yes, regularly □ Yes, occasionally □ No, not in the stage of extensive disease
34. Do you see any problems with cranial irradiation of brain metastases in the context of therapy with atezolizumab? □ Radiation and simultaneous treatment with atezolizumab are generally possible □ A minimum interval of one day should be observed □ A minimum interval of one week should be observed □ A minimum interval of more than one week should be observed

*SCLC* small-cell lung cancer, *LS-SCLC* limited stage small-cell lung cancer, *ES-SCLC* extensive stage small-cell lung cancer, *PCI* prophylactic cranial irradiation, *GTV* macroscopic tumor volume, *CTV* clinical target volume, *NYHA* New York Heart Association, *FEV1* forced expiratory pressure in 1 second, *EBUS* endobronchial ultrasoundeGFR estimated glomerular filtration rate, *CT* computed tomography, *MRI* magnetic resonance imaging, *PET-CT* positron emission tomography, *Gy* gray

## Abbreviations

BID: *Bis in die* (twice a day)
CI: Confidence interval
CRT: Chemoradiotherapy
CTV: Clinical target volume
ECOG: Eastern Cooperative Oncology Group
ES: Extensive stage
ES-SCLC: Extensive disease small-cell lung cancer
FDG: Fluorodeoxyglucose
GTV: Macroscopic tumor volume
HR: Hazard ratio
LC: Limited stage
LRC: Locoregional control
LS-SCLC: Limited stage small-cell lung cancer
MTV: Metabolic tumor volume
NSCLC: Non-small-cell lung cancer
PCI: Prophylactic cranial irradiation
PD-L1: Programmed death ligand 1
PFS: Progression-free survival
OR: Odds ratio
OS: Overall survival
RCT: Randomized controlled trial
RT: Radiotherapy
SCLC: Small-cell lung cancer
SUV_max_: Maximum standardized uptake value
TRAE: Treatment-related adverse events
